# Allele Loss and Down-Regulation of Heparanase Gene Are Associated with the Progression and Poor Prognosis of Hepatocellular Carcinoma

**DOI:** 10.1371/journal.pone.0044061

**Published:** 2012-08-31

**Authors:** Guo-Liang Huang, Bin-Kui Li, Mei-Yin Zhang, Rong-Rong Wei, Yun-Fei Yuan, Ming Shi, Xiao-Qian Chen, Long Huang, Hui-Zhong Zhang, Wanqing Liu, Bi-Jun Huang, Honghua Li, Xiao-Feng Zheng, Xian-Rong Luo, Hui-Yun Wang

**Affiliations:** 1 National Key Laboratory of Oncology in South China, Sun Yat-Sen University Cancer Center, Guangzhou, Guangdong, China; 2 Sino-American Cancer Research Institute, Guangdong Medical College, Dongguan, Guangdong, China; 3 Department of Hepatobiliary Oncology, Sun Yat-Sen University Cancer Center, Guangzhou, Guangdong, China; 4 Department of Pathology, Sun Yat-Sen University Cancer Center, Guangzhou, Guangdong, China; 5 Section of Hematology/Oncology, Department of Medicine, University of Chicago, Chicago, Illinois, United States of America; 6 Department of Pharmacology, Robert W. Johnson Medical School, UMDNJ, Piscataway, New Jersey, United States of America; 7 The 458 Hospital of PLA, Guangzhou, Guangdong, China; Mount Sinai School of Medicine, United States of America

## Abstract

**Objectives:**

The role of heparanase (HPSE) gene in cancers including hepatocellular carcinoma (HCC) is currently controversial. This study was aimed at investigating the impact of genetic alteration and expression change of HPSE on the progression and prognosis of HCC.

**Methods:**

The HPSE gene was studied in three different aspects: (1) loss of heterozygosity (LOH) by a custom SNP microarray and DNA copy number by real-time PCR; (2) mRNA level by qRT-PCR; and (3) protein expression by immunohistochemistry. The clinical significances of allele loss and expression change of HPSE were analyzed.

**Results:**

Microarray analysis showed that the average LOH frequency for 10 SNPs located within HPSE gene was 31.6%, three of which were significantly correlated with tumor grade, serum HBV-DNA level, and AFP concentration. In agreement with SNP LOH data, DNA copy number loss of HPSE was observed in 38.74% (43/111) of HCC cases. HPSE mRNA level was notably reduced in 74.1% (83/112) of tumor tissues compared with non-tumor liver tissues, which was significantly associated with DNA copy number loss, increased tumor size, and post-operative metastasis. HPSE protein level was also remarkably reduced in 66.3% (53/80) of tumor tissues, which was correlated with tumor grade. Patients with lower expression level of HPSE mRNA or protein had a significantly lower survival rate than those with higher expression. Cox regression analysis suggested that HPSE protein was an independent predictor of overall survival in HCC patients.

**Conclusions:**

The results in this study demonstrate that genetic alteration and reduction of HPSE expression are associated with tumor progression and poor prognosis of HCCs, suggesting that HPSE behaves like a tumor suppressor gene and is a potential prognostic marker for HCC patients.

## Introduction

Heparanase (HPSE) is an endoglycosidase that cleaves side chains of heparan sulfate (HS), a linear polysaccharide found on the cell surface and extracellular matrix (ECM), which plays critical roles in cell-cell and cell-matrix interactions [1]. HS also tethers a multitude of growth factors, chemokines, cytokines and enzymes to the ECM and cell surface [2]. Hence, HPSE not only participates in degradation and remodeling of the ECM, but also releases HS-bound biological molecules by cleavage of HS side chains [3]. Aside from the well-studied catalytic features of the enzyme, non-enzymatic functions of HPSE include enhancement of cell adhesion [3] and inducing phosphorylation of p38 [4], Akt [5] and VEGF [6]. Altogether, HPSE may have extensive and complex effects on wide variety of biological activities.

Due to its important and extensive biological activities, HPSE also plays a critical role in cancer development and progression. Many studies have shown that HPSE is up-regulated in a variety of primary human tumors, which is correlated with higher incidence of lymph node and distant metastasis, increased micro-vessel density and reduced post-operation survival of cancer patients [7], [8]. These studies suggested that HPSE behaves like an oncogene or tumor promoter. However, other studies showed opposite results. For example, studies on clinical tumor samples indicated that the up-regulated HPSE in the cell nucleus was correlated with a favorable outcome in patients with esophageal squamous cell carcinomas [9], gastric carcinomas [10], head and neck carcinomas [11] and lung cancer [8]. Conflicting results were also reported in hepatocellular carcinoma (HCC) [12]. Therefore, it remains unclear whether PHSE is a suppressor or promoter of human cancers, especially for HCC [12], which is possibly related to the extensive and complex functions of HPSE.

**Table 1:** LOH frequency of 10 SNPs in HPSE gene at D4S2964 on Chromosome 4q21.

SNP ID	Gene region	Physical location (bp)	Case No of LOH	Case No of Retention	LOH Frequency
rs9991877	intron 11	8764809	5	30	0.143
rs4364254	intron 9	8771434	14	15	0.483
rs11099592	exon 7	8778340	6	11	0.353
rs6535455	intron 4	8779825	5	22	0.185
rs6535458	intron 4	8781170	6	12	0.333
rs7691732	intron 3	8784245	9	11	0.450
rs6535462	intron 3	8786233	8	16	0.333
rs4568236	intron 3	8787803	3	10	0.231
rs12501123	intron 2	8788375	3	7	0.300
rs4693611	intron 1	8795441	8	14	0.364

In a previous study, we performed a genome-wide analysis of loss of heterozygosity (LOH) in 104 HCCs with 382 microsatellite markers and found that the LOH rate of D4S2964 on 4q21.1 was as high as 50% [13]. This result was consistent with Bando's report in 1999, which found this locus with 41.5% LOH in HCC [14], and Nishimura's study in 2006, which reported that a deletion region containing D4S2964 occurred in 47% of HCC patients [15]. In addition, other genetic studies on HCC showed that chromosome 4q21, where the D4S2964 locus was located, was a common deleted region in HCC [16], [17]. All of these evidences indicated that the D4S2964 locus might contain a tumor suppressor gene(s) in HCC. In order to identify the gene(s) involved in this LOH region, we performed a fine-scale LOH analysis with 440 SNP markers located in 49 genes surrounding D4S2964 locus in 112 paired HCC and adjacent non-tumor liver tissues using a custom SNP microarray, and found a high frequency of LOH in HPSE gene [18]. Our results suggest that HPSE is a tumor suppressor gene based on the fact that tumor suppressor gene usually has LOH in carcinogenesis. Combined with observations from our previous study and by others, we hypothesized that HPES was a tumor suppressor gene in HCC. To support the tumor suppressor role of this gene, we further investigated the genetic alterations and expression changes of the HPSE gene in HCCs and evaluated their clinical implications. Our results show that the allele loss and reduced HPSE expression are indeed closely correlated with tumor progression and poor prognosis of HCC patients.

**Figure 1:**
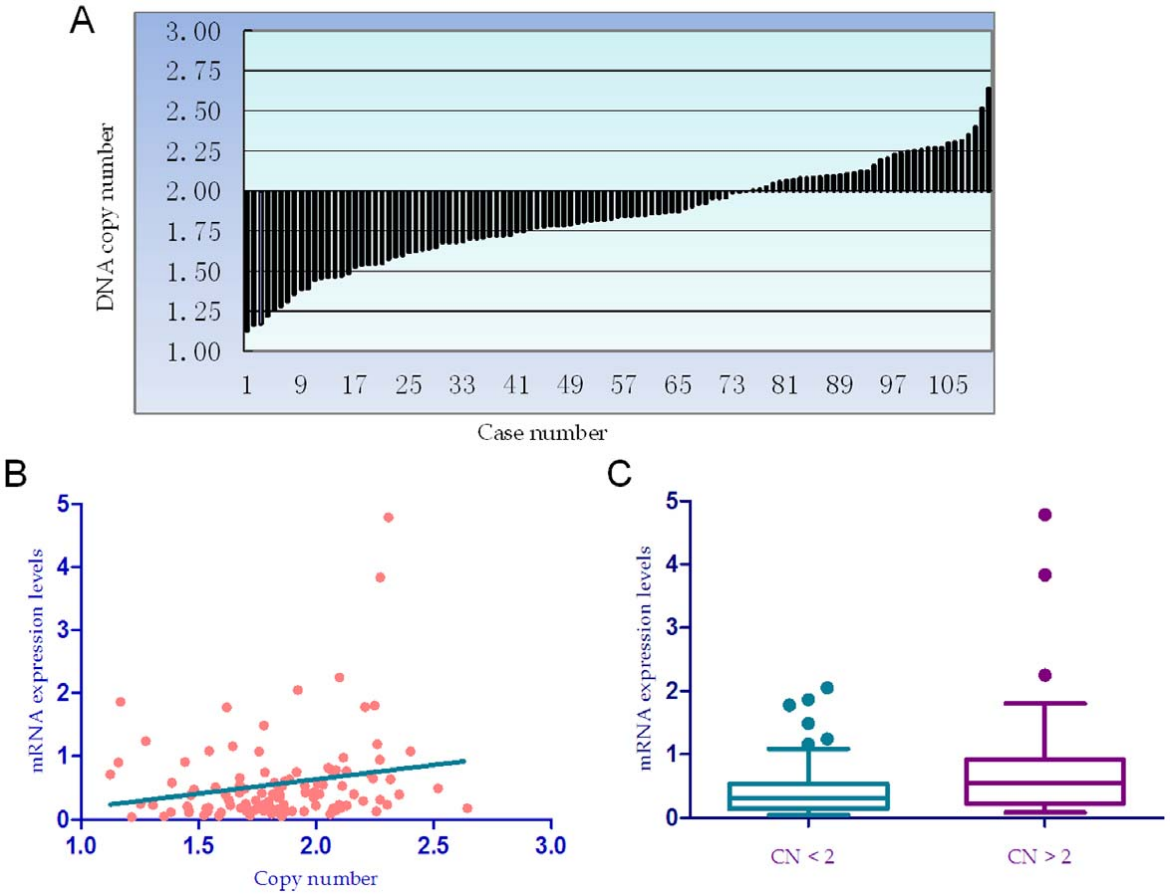
HPSE DNA copy number variation and correlation with mRNA expression in HCC. (a), HPSE DNA copy number in 111 HCC samples. The normal range was 1.76–2.24, which was normalized by the normal liver tissues. (b), Correlation between HPSE DNA copy number and mRNA expression levels in 111 HCC samples, r = 0.242, *P*<0.028. (c), The mRNA expression level of HPSE in HCC patients with HPSE DNA copy number <2 was significantly lower than that in those with DNA copy number >2, *P* = 0.005.

## Methods

### Patients and tissue samples

All 112 patients with HCC received hepatectomy between 2004 and 2007 at the Department of Hepatobiliary Oncology at the Sun Yat-Sen University Cancer Center. HCC and corresponding non-tumor liver tissues were collected at the time of hepatectomy. There were about 1563 patients with HCC undergoing hepatectomy between 2004 and 2007 in the cancer center. However, only patients who met all of the following criteria were included in this study: 1) did not receive any other anti-cancer therapies before the surgery, such as chemoembolization, chemotherapy, etc; 2) underwent curative resection for complete removal of the tumor without macroscopic evidence of residual cancer tissues; 3) diagnosed with HCC by pathology; 4) had frozen tumor tissues available in the Tissue Bank of the cancer center. The 112 HCC patients included 94 males and 18 females with a median age of 45.5 years (range, 13–72 years). No patients had extrahepatic metastasis when they underwent hepatectomy. Postoperative metastasis meant the extrahepatic metastasis to distant organs and recurrence was the intrahepatic recurrence after hepatectomy. The patients were followed up every 2 to 4 months in the first two years, and thereafter annually, with a median follow-up time of 36 months. Patients who returned to the hospital were detected by Computed tomography (CT) or Ultrasound B-mode scanner and AFP test in the follow-up or contacted by mail or call. The primary endpoint was overall survival from the date of hepatectomy to patient death or the last follow-up. Written informed consent was obtained from all the participants or guardians on the behalf of the children participants. This study was reviewed and approved by the Committee for the Conduct of Human Research of the Sun Yat-Sen University Cancer Center.

**Figure 2:**
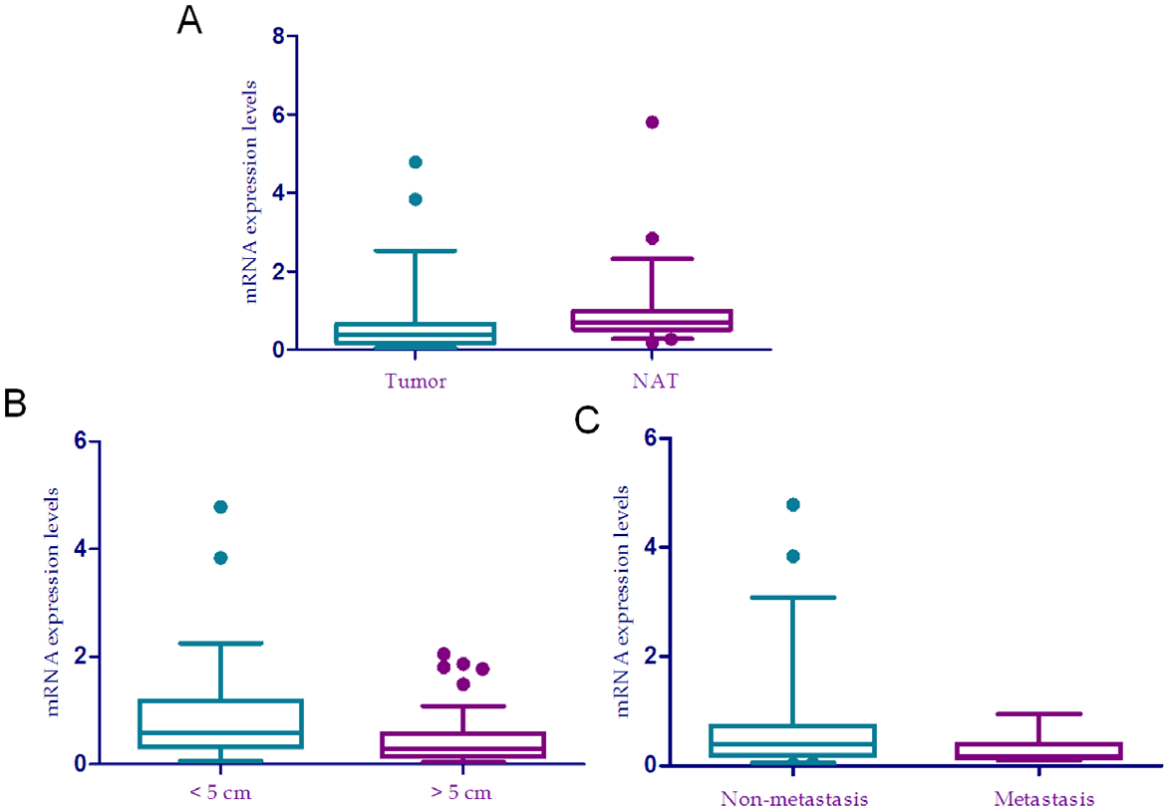
Relative expression level of HPSE mRNA in HCC samples with different clinical features. (a), mRNA level in HCC (Tumor) was lower than that in corresponding non-tumor tissues (NAT), *P* = 0.0005. (b), mRNA level in tumors with size >5 cm was lower than that in tumors with size ≤5 cm, *P* = 0.0015. (c), mRNA in patients with post-operative metastasis was lower than that in those without, *P* = 0.0336.

The fresh samples were immediately immersed in RNAlater (Ambion, Inc., USA) after surgical resection, and stored at 4°C overnight to allow thorough penetration of the tissues. The samples were then frozen at −80°C until RNA and DNA extraction. Total RNA and DNA were extracted sequentially using TRIzol reagent (Invitrogen, USA) according to the manufacturer's instructions.

**Figure 3:**
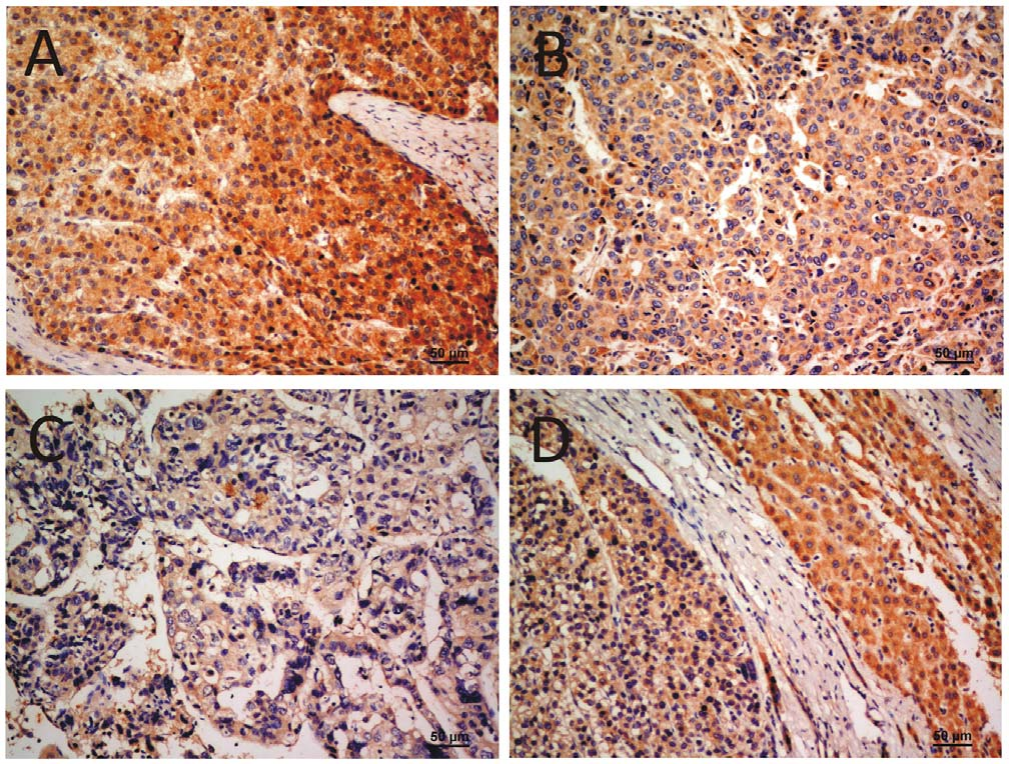
Immunohistochemical detection of the HPSE protein expression in HCC. The tissue sections were developed with DAB and counterstained by hematoxylin. (a), Well differentiated hepatocellular carcinoma (200×), HPSE staining was scored as positive 3; (b), moderately differentiated hepatocellular carcinoma (200×), scored as positive 2; (c), poorly differentiated hepatocellular carcinoma (200×), scored as positive 1; (d), Immunostaining of HCC and adjacent surrounding non-cancerous liver tissues, HCC part (left and below) with weak staining, and the adjacent liver tissue with strong staining (200×).

### SNP genotyping by microarray and data processing

The procedure of selecting 440 SNPs in 49 genes was described by Huang et al [18]. The polymorphic sites of the SNPs were all transition variations (A/G or C/T) to facilitate microarray analysis by using two fluorescent colors (Cy-3 and Cy-5). The SNP microarray genotyping system described by Wang HY *et* *al* [19] was used with minor modifications. The microarray consisting of probes for the 440 SNPs was printed on cleaned slides by a SmartArray^TM^-136 printer, hybridized with the amplified single-strand DNAs containing the SNP sites in a BioMixer^TM^ II hybridization oven and scanned by a LuxScan^TM^-10 K Scanner (CapitalBio Inc., Beijing, China). Scanned images of microarrays were analyzed using GenPix Pro 6.0 software (Axon Instruments, Foster City, CA, USA). The hybridization signals were presented as median intensity for each feature. Data normalization, low-signal filtering, background subtraction and genotyping were processed by our developed SNP genotype analysis program called “AccuTyping” [20]. Genotype call for each SNP was determined based on the signal intensity ratio of the two fluorescences [18].

**Table 2:** Correlation of HPSE expression with clinicopathological features.

	HPSE mRNA level			HPSE protein score		
Parameter	Low mRNA	High mRNA	*P* value	Low score	High score	*P *value
	N (%)	N (%)		N (%)	N (%)	
Sex						
Female	10 (18.2)	8 (14.0)	0.55	6 (10.5)	7 (23.3)	0.126
Male	45 (81.8)	49 (86.0)		51 (89.5)	23 (76.7)	
Age						
<50	37 (67.3)	34 (59.6)	0.402	36 (63.2)	18 (60.0)	0.773
≧50	18 (32.7)	23 (40.4)		21 (36.8)	12 (40.0)	
Serum HBsAg						
Negative	9 (16.4)	7 (12.3)	0.537	6 (10.5)	6 (20.0)	0.326
Positive	46 (83.6)	50 (87.7)		51 (89.5)	24 (80.0)	
Serum HBV-DNA						
Negative	29 (58.0)	34 (65.4)	0.443	28 (52.8)	17 (63.0)	0.388
Positive	21 (42.0)	18 (34.6)		25 (47.2)	10 (37.0)	
Serum HD-Ag						
Negative	53 (96.4)	57 (100)	0.239	56 (98.2)	29 (96.7)	0.64
Positive	2 (3.6)	0 (0)		1 (1.8)	1 (3.3)	
Serum HBeAg						
Negative	49 (89.1)	50 (87.7)	0.821	46 (80.7)	28 (93.3)	0.204
Positive	6 (10.9)	7 (12.3)		11 (19.3)	2 (6.7)	
Cirrhosis						
Absent	3 (6.1)	9 (17.6)	0.076	6 (11.5)	4 (16.0)	0.72
Present	46 (93.9)	42 (82.4)		46 (88.5	21 (84.0)	
Tumor size (cm)						
≤5	6 (10.9)	19 (33.3)	0.004	16 (28.1)	6 (20.0)	0.41
>5	49 (89.1)	38 (66.7)		41 (71.9)	24 (80.0)	
No. of nodules						0.889
1	40 (72.7)	43 (75.4)	0.743	41 (71.9)	22 (73.3)	
>1	15 (27.3)	14 (24.6)		16 (18.1)	8 (16.7)	
Tumor grade*						
Grade I	7 (13.2)	2 (3.6)	0.188	2 (3.5)	6 (20.7)	0.012
Grade II	35 (66.0)	41 (73.2)		39 (68.4)	20 (69.0)	
Grade III	11 (20.8)	13 (23.2)		16 (28.1)	3 (10.3)	
Post-operative Metastasis						
No	45 (81.8)	53 (93.0)	0.074	49 (86.0)	28 (93.3)	0.306
Yes	10 (18.2)	4 (7.0)		8 (14.0)	2 (6.7)	
Recurrence						
No	32 (58.2)	43 (75.4)	0.052	34 (59.6)	24 (80.0)	0.056
Yes	23 (41.8)	14 (24.6)		23 (40.4)	6 (20.0)	

HBsAg: the surface antigen of the hepatitis B virus; HBV-DNA: hepatitis B virus DNA; HD-Ag: hepatitis D antigen; HBeAg: hepatitis B e-antigen.

* Tumor grade: evaluated with Edmondson-Steiner grading system, and no cases was in grade IV.

### LOH analysis

The purpose of SNP genotyping in this study was for LOH analysis. If a SNP was typed as heterozygous in non-tumor tissue of a patient, this SNP in the patient was defined as informative SNP. Only informative SNPs were included in LOH analysis. SNP LOH was defined when a SNP was heterozygous in a non-tumor liver tissue and homozygous in the corresponding HCC tissue. If a SNP was heterozygous in both paired non-tumor liver and tumor tissue, it was defined as retention. Frequency of SNP LOH for each SNP site was equal to the ratio of the number of cases with the SNP LOH to the number of cases with the informative SNPs for a SNP in all of the cases. If any one or more SNPs had LOH in the HPSE gene in one case, it was defined as Gene LOH in this case. Informative case for a gene was defined as a case with any informative SNPs in this gene. Frequency of Gene LOH for a gene was equal to the ratio of the number of cases with the Gene LOH to the number of cases with informative cases.

**Figure 4:**
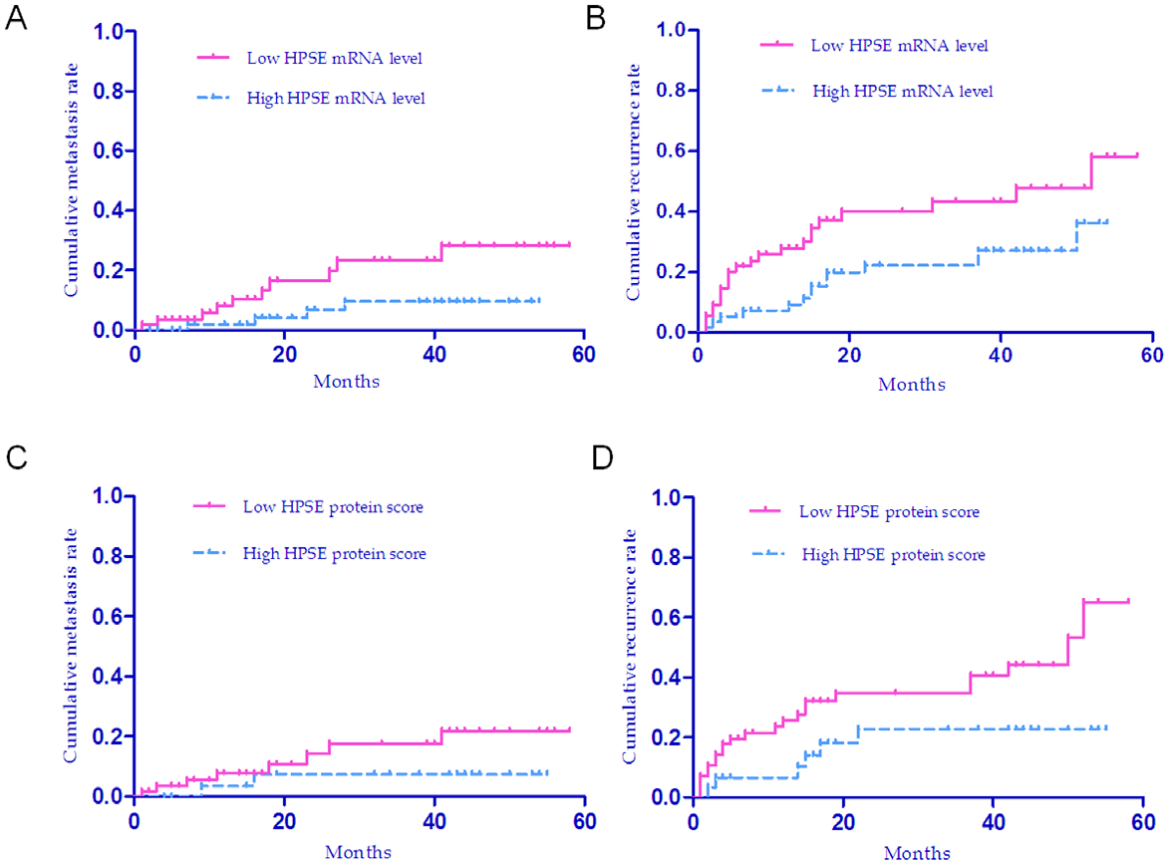
Cumulative metastasis rate and recurrence rate of HCC patients with high and low expression levels of HPSE analyzed by Kaplan–Meier Curve. (a), Cumulative metastasis curves for patients with high or low HPSE mRNA levels (Log-rank test, *P* = 0.033). (b), Cumulative recurrence curves for patients with high or low HPSE mRNA levels (Log-rank test, *P* = 0.023). (c), Cumulative metastasis curves for patients with high or low HPSE protein staining scores (Log-rank test, *P* = 0.199). (d), Cumulative recurrence curves for patients with high or low HPSE protein staining scores (Log-rank test, *P* = 0.35).

**Table 3:** Multivariate Cox regression analysis of impacts of variables affecting recurrence.

Parameter	Hazard ratio	Confidence interval (95%)	*P* value
HPSE mRNA level*	2.332	1.083–5.021	0.031
Serum AFP	2.684	1.019–7.072	

* In univariate analysis, both mRNA and protein of HPSE were significant for recurrence. In this multivariate analysis, only mRNA level with smaller p value was included.

### Quantitative PCR (qPCR)

HPSE DNA copy number quantification by qPCR described by Liu et al [21] was performed using Platinum SYBR Green qPCR SuperMix-UDG reagents (Invitrogen, USA) in Applied Biosystems PRISM 7900HT instruments. Briefly, a 142-bp amplicon of HPSE was amplified with a pair of primers (forward: 5′-GTT TGG CTT TGA GCT TTG CTT-3′ and reverse: 5′- ATC GTG CTT GCT GCT TTT TAT C-3′). Another 149-bp amplicon of the LINE1 sequence was used as an internal control [22]. Real-time qPCR was performed in a 15-μl reaction mixture containing 10 ng genomic DNA, 0.25 μl of 10 mM each primer (forward and reverse primer) and 7.5 μl of 2× SYBR Green PCR Master Mix. The PCR was cycled 45 times at 95°C for 30 s, 60°C for 1 min, after preheating at 95°C for 10 min. All reactions were run in duplicate. The HPSE copy number was normalized to that of LINE1 to obtain a ratio (R1) for each tissue sample. Sample R1 was then normalized to the average R1 of 10 normal liver samples (the samples were obtained from the normal liver tissues of the resected edges of hepatoadenomas, none of them had hepatitis B) to get the second ratio (R2), and the HPSE copy number for each tissue was calculated by doubling R2. Based on the values of normal liver tissues, copy number change between 1.76 and 2.24 would be considered as normal, less than 1.76 defined as a DNA copy number loss and greater than 2.24 defined as a DNA copy number gain.

**Figure 5:**
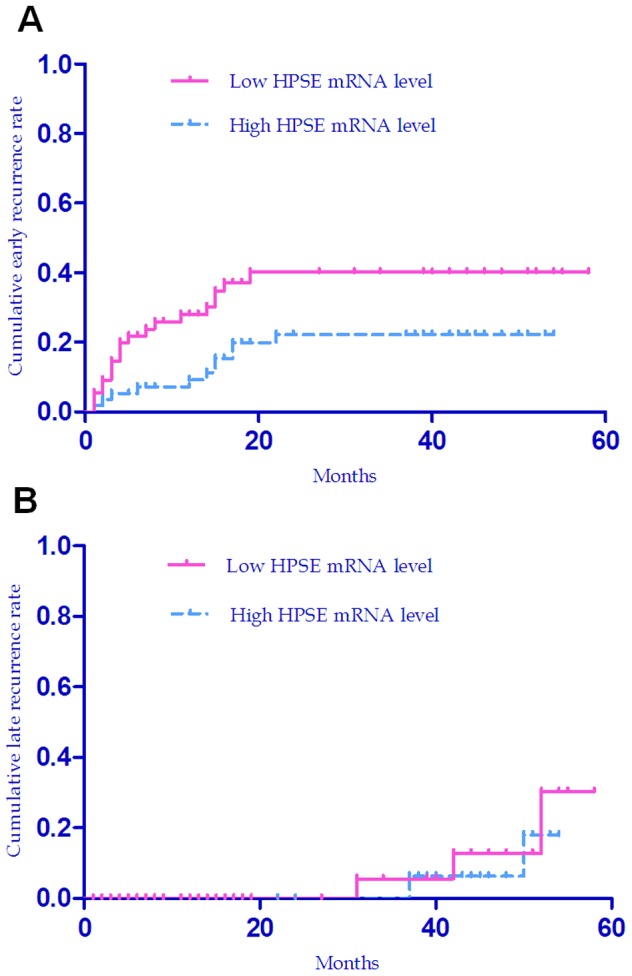
Cumulative early and late recurrence rate of HCC patients with high or low expression levels of HPSE by Kaplan–Meier Curve. (a), Cumulative early recurrence (within 2 years) curves for patients with high or low HPSE mRNA levels (Log-rank test, *P* = 0.025). (b), Cumulative late recurrence (in more than 2 years) curves for patients with high or low HPSE mRNA levels (Log-rank test, *P* = 0.582).

### Quantitative RT-PCR (qRT-PCR)

Reverse transcription reaction was carried out with MMLV reverse transcription kit according to the manufacturer's protocol (Promega, USA). Two micrograms of total RNA were used for reverse-transcription reaction to generate cDNA at 42°C for 60 min. Then qPCR assay was performed with Platinum SYBR Green qPCR SuperMix-UDG reagents (Invitrogen, USA) in Applied Biosystems PRISM 7900HT instruments according to the manufacture's protocol. The reactions with 0.5 μl of cDNA, 7.5 μl of 2× SYBR Green qPCR SuperMix, 0.25 μl each of 10 mM forward and reverse primers at 15 μl volume were carried out in a 96-well plate at 95°C for 10 min, followed by 40 cycles of 95°C for 30 sec and 60°C for 1 min. Each sample was run in duplicate. The primers for 11 genes with higher LOH and a housekeeping gene, glyceraldehyde-3-phosphate dehydrogenase (GAPDH), were listed in Table S1. The comparative Ct method (ΔΔCt) was used for quantification of the HPSE gene expression and relative quantification (RQ) was calculated as 2^−ΔΔCt^.

**Table 4:** Multivariate Cox regression analysis of variables affecting early recurrence.

Parameter	Hazard ratio	Confidence interval (95%)	*P* value
HPSE mRNA level	2.401	1.146–5.028	0.020
Serum AFP	3.830	1.337–10.971	0.012

### Immunohistochemistry (IHC)

Formalin-fixed and paraffin-embedded tissues were cut into 4-μm sections and mounted onto the polylysine-coated slides. After treated with routine procedures, the sections were incubated for 1 hour at 37°C with rabbit anti-HPSE polyclonal antibody (Santa Cruz Biotechnology, Inc., USA) diluted 1∶200 in blocking solution followed by another reaction with HRP-conjugated secondary antibody (ChemMate Envision Detection Kit, Dako) according to the manufacturer's instruction. After washes, color was developed with 3,3′-diaminobenzidine tetrahydrochloride (DAB), and then all of the sections were counterstained with hematoxylin. For negative controls, tissue sections were incubated without anti-HPSE antibody under the same experimental conditions. HPSE staining was scored according to its intensity (0, no staining; 1, weak staining; 2, moderate staining; 3, strong staining) and the percentage of tumor cells that were stained (0, <5% of tumor cells stained; 1, 5%–25% of tumor cells stained; 2, 25–50% of tumor cells stained; 3, >50% of tumor cells stained). The final expression score was calculated from ‘intensity score’ multiplied by ‘percentage’: I stands for scores 0–1, II for scores 2–3, III for scores 4–6 and IV for scores >6. For statistical analysis, we combined the cases scored as I and II (low score) to compare with the cases with scored as III and IV (high score). Immunohistochemistry analysis was performed on 80 available cases of the 112 patients.

**Figure 6:**
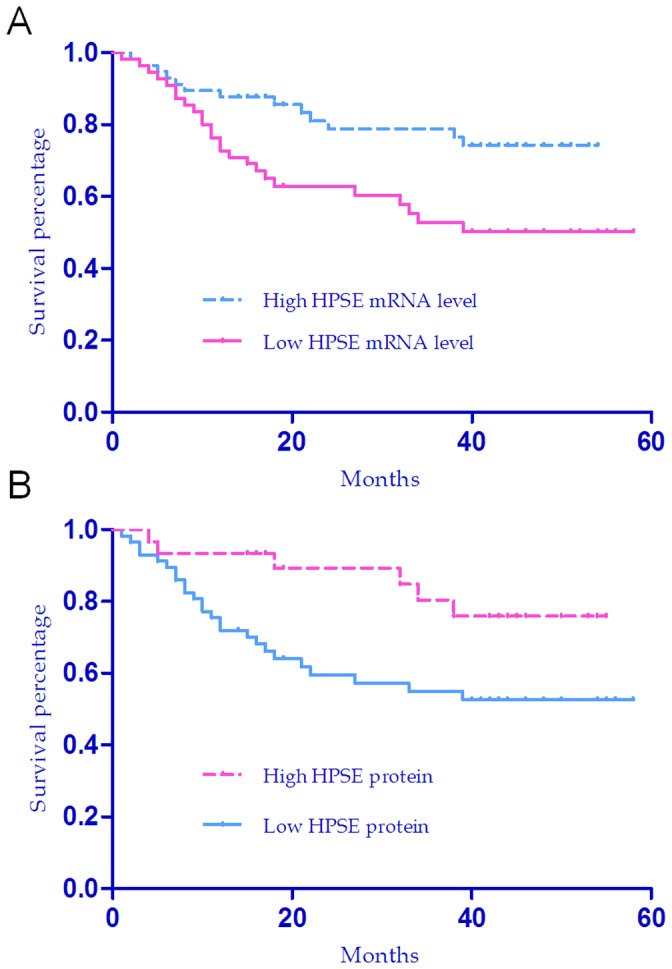
Overall survival curves of HCC patients with high or low expression levels of HPSE by Kaplan–Meier Curve. (a), Overall survival curves by HPSE mRNA levels (Log-rank test, *P* = 0.009). (b), Overall survival curves by HPSE protein staining scores (Log-rank test, *P* = 0.024).

### The process for identification of genes that might be affected by LOH

In order to identify the genes involved in the LOH of D4S2964, all of 49 genes in this locus were detected by SNP microarray and analyzed by LOH method. In this step, the LOH frequency for all of the genes in the locus would be determined. If a gene has LOH, the mRNA level of this gene usually will be reduced. Thus, the mRNA level of the genes in the top-10 list of LOH and another gene were measured by qRT-PCR in 50 pairs of HCC and corresponding non-tumor liver tissues, and the relationships between the mRNA levels of the 11 genes and patient's survival were analyzed by Kaplan-Meier curve and Log-rank test. The genes that were significantly associated with poor survival (P<0.1) were further investigated by qRT-PCR and Kaplan-Meier curve with Log-rank test in all of the 112 HCC cases. If any gene still was correlated with poor survival of the 112 patients, it would be selected for further study.

**Table 5:** Multivariate Cox regression analysis of variables affecting overall survival.

Parameter	Hazard ratio	Confidence interval (95%)	*P* value
HPSE protein score*	2.567	1.004–6.561	0.049
Tumor grade	1.180	0.763–1.825	0.457
Serum AFP	2.090	0.673–6.487	0.202
Tumor size	3.929	1.117–13.819	0.033
No. of nodules	2.843	1.310–6.168	0.008

* In univariate analysis, both mRNA and protein of HPSE were significant for overall survival. In this multivariate analysis, only protein score with smaller p value was included.

**Table 6:** Multivariate Cox regression model analysis of variables affecting overall survival in the subgroup of BCLC stage B,C,D.

Parameter	Hazard ratio	Confidence interval (95%)	*P* value
HPSE protein score	6.398	1.337–30.613	0.020
Tumor grade	2.489	0.923–6.709	0.071

### Statistical analysis

The chi-square test, Fisher's exact test and Mann-Whitney *U* test were used to analyze the correlations between clinicopathological features with the HPSE LOH status, DNA copy number or expressions. The overall survivals, cumulative metastasis rates and cumulative recurrence rates in different groups were estimated using the Kaplan-Meier analysis and Log-rank test. In order to identify the parameters (clinicopathological features and HPSE expressions) that might affect the survival or metastasis or recurrence, we first performed univariate Cox regression analysis on all of parameters. Then, those parameters would be moved into the multivariate Cox regression analysis with an Enter procedure if they displayed statistical significance (P<0.05) in the univariate analysis. The probability for stepwise variable selection was set at 0.05 for entry and 0.10 for removal. The SPSS version 16.0 software package and GraphPad Prism were used for the statistical analysis and data plotting.

**Figure 7:**
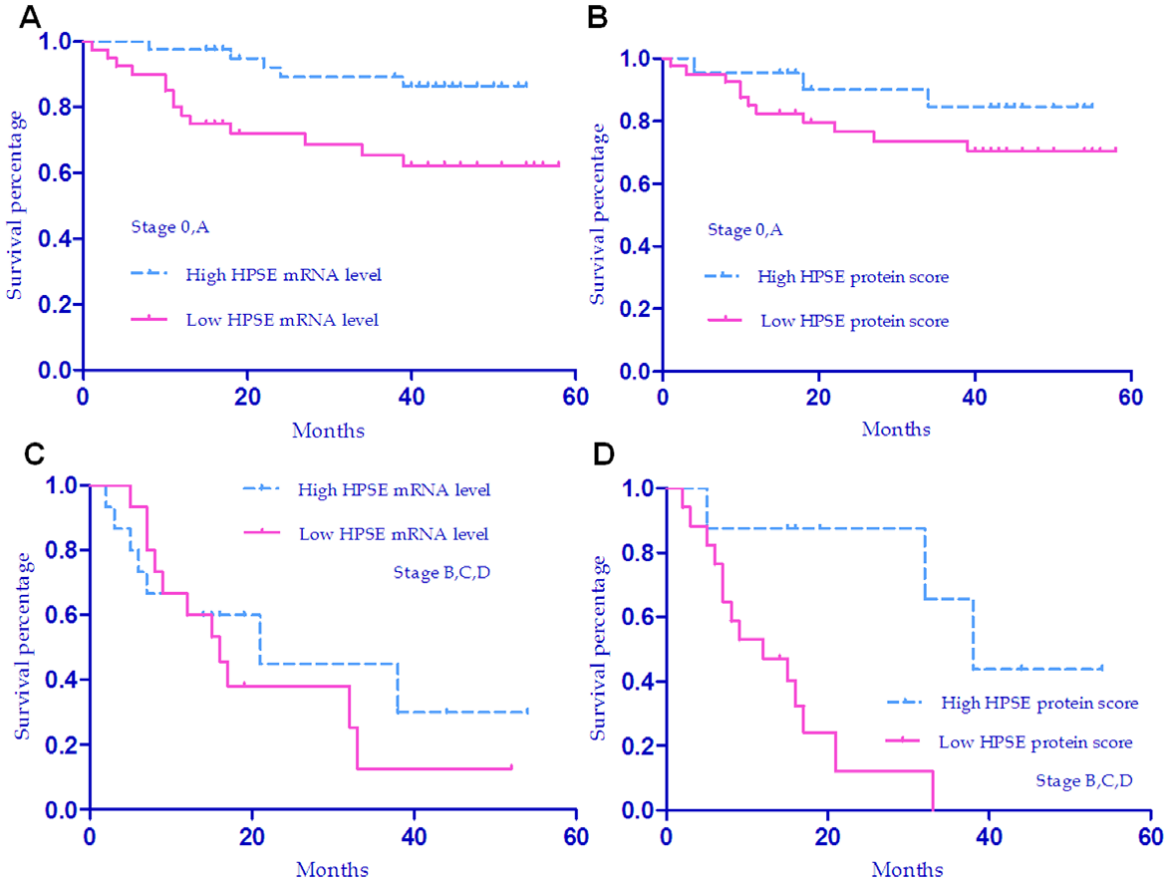
Overall survival curves of HCC patients with high or low expression levels of HPSE in the subgroups of BCLC stages. (a), Overall survival curves by HPSE mRNA levels in the subgroup of BCLC stage 0,A (Log-rank test, *P* = 0.008). (b), Overall survival curves by HPSE protein staining scores in the subgroup of BCLC stage 0,A (Log-rank test, *P* = 0.214). (c), Overall survival curves by HPSE mRNA levels in the subgroup of BCLC stage B,C,D (Log-rank test, *P* = 0.502). (d), Overall survival curves by HPSE protein staining scores in the subgroup of BCLC stage B,C,D (Log-rank test, *P* = 0.004).

## Results

### Analysis of SNP LOH and mRNA level suggests that HPSE is involved in the LOH of D4S2964

A total of 13,322 genotypes out of 49,280 SNPs were determined as heterozygous in 112 non-tumor liver tissues for the 440 SNPs in the 49 genes surrounding D4S2964 locus, and 25 genotypes of them were verified by sequencing [18]. This microarray data and related information had been deposited into Gene Expression Omnibus (GEO) database (http://www.ncbi.nlm.nih.gov/geo/). The accession number is GSE36277. In the LOH analysis of SNPs, only cases (informative) with heterozygous SNP in non-tumor liver tissue were included. In the present study, the number of informative cases for each SNP ranged from 0 to 65 with an average of 24.2. Sixty-three SNPs located in 32 genes had a LOH frequency greater than 30%, and LOH of the 49 genes had been reported elsewhere [18].

In general, if a gene had LOH, its mRNA expression would be reduced. Thus, we selected 10 genes (PPEF2, PRDM8, SDAD1, CXCL9, CCDC158, PRKG2, HPSE, AGPAT9, HELQ, NUP54) with the highest frequency of Gene LOH (Table S2) and another gene (CCNG2) based literature reports, which suggested CCNG2 as a tumor suppressor, to detect mRNA expression level in 50 pairs of HCC and the matched liver samples from the 112 cases. The result indicated that the expression level of most genes was much lower in tumors than in the matched non-tumor liver tissues (Table S2). In order to identify key gene(s) in this LOH region, we conducted a correlation analysis on the mRNA expression level of the 11 genes and patients’ prognosis. The result indicated that mRNA expression level for five (PPEF2, CCDC158, CCNG2, HPSE, AGPAT9) of these genes was significantly associated with patients’ prognosis in the 50 HCC cases (Table S2). Then, mRNA expression level for the five genes was further measured in the others of the 112 HCC patients, and survival analysis of the 112 cases was performed on the mRNA expression levels. The result showed that the low expression levels of four out of the five genes were associated with poor survival of HCC patients (Table S2). Based on the above results, we decided to further investigate the clinical significances and/or functions of the four genes (CCDC158, CCNG2, HPSE and AGPAT9). Some of this work are still ongoing. HPSE was one of the four genes. These data implied that HPSE is a tumor suppressor gene, which was contradictory with the notion that HPSE is an oncogene or tumor promoter. Therefore, it is necessary to further investigate this gene and its clinical significance in HCC because it should help to clarify its clinical significance, which is of obvious importance to the medical field, especially to HCC biology and therapy.

### The high frequency of SNP LOH in HPSE is associated with serum AFP, HBV-DNA and tumor grade

Of the 12 SNPs located in the HPSE gene, 10 were genotyped as heterozygous in 10 or more cases (informative cases) among the 112 samples. The average LOH frequency for the 10 SNPs was 31.6% with a range from 14.3% (5/35 for rs9991877) to 48.3% (14/29 for rs4364254) (Table 1). Correlation analysis between the LOH frequency of each SNP in HPSE gene and clinical features (serum AFP level, serum HBV-DNA level, HBsAg status, HBeAg status, tumor grade, tumor size, metastasis and recurrence) revealed that LOHs at three SNPs, rs4364254, rs6535458 and rs4568236, were significantly correlated with serum AFP level, tumor grade and serum HBV-DNA level (all *P*<0.05), respectively. Out of 84 informative cases, 41 (48.8%) had Gene LOH (see LOH analysis in Method). However, no significant correlation was found between the Gene LOH and clinicopathological features.

### HPSE DNA copy number loss in HCC is correlated with SNP LOH and mRNA level

Real-time PCR is a common and efficient method for measuring DNA copy number. In the present study, we employed real-time PCR to detect the HPSE DNA copy number alteration. In 111 HCCs with successful detection, the median of DNA copy number was 1.84 with a range from 1.12 to 2.64. DNA copy number loss was observed in 38.74% (43/111) of tumor tissues, and copy number gain in 12.61% (14/111) (Fig 1A). The correlation analysis showed that DNA copy number and Gene LOH were significantly associated in 84 cases (r = 0.242, *P*<0.028). To explore the correlation between HPSE DNA copy number and mRNA expression, HPSE mRNA was quantified by real-time RT-PCR. The result showed a small but significant correlation between HPSE copy number and mRNA levels in tumor tissues (r = 0.23, *P* = 0.013, Fig 1B). The HPSE mRNA levels in HCC tissues with DNA copy number less than 2 were significantly lower than those with DNA copy number more than 2 (*P* = 0.0055, Fig 1C).

### Reduced HPSE mRNA level is related with tumor size and metastasis in HCC

We next compared mRNA expression levels between paired HCC samples and non-tumor liver tissues, and found that the HPSE mRNA level in HCC was significantly lower than that in the paired non-tumor liver tissues (*P* = 0.0005, Fig. 2A). The mRNA level of HPSE in 83 (83/112 = 74.1%) cases was reduced in tumor tissues compared with non-tumor liver tissues. To investigate the significance of the reduced HPSE mRNA level in HCC progression, we performed a correlation analysis with clinicopathological parameters. The result indicated that HPSE mRNA expression was significantly reduced in tumors with greater than 5 cm compared with tumors equal to or smaller than 5 cm (*P* = 0.0015, Fig. 2B), and in patients with post-operative metastasis compared with patients without (*P* = 0.0336, Fig. 2C). No correlation was found between HPSE mRNA level and other clinical features by this analysis.

### Down-regulation of HPSE protein in HCC is correlated with tumor grade

In general, the reduced mRNA expression of a gene will cause down-regulation of corresponding protein. To confirm this situation in HCC, HPSE protein in 80 cases of the 112 HCCs were detected by IHC. The result exhibited a lower staining score of HPSE protein in 53 (66.3%) HCC tissues than in their matched non-tumor adjacent tissues (Fig 3). Interestingly, HPSE protein was detected in both the cytoplasm and nucleus of liver cancer cell (Fig 3), which is in sharp contrast to previous studies that found HPSE protein exclusively in either the cytoplasm or nucleus of cancer cells [8], [9], [10], [11]. The χ^2^ test indicated that the staining score of HPSE protein was significantly correlated with tumor grade (*P* = 0.012, Table 2), and that the correlation between HPSE protein expression and recurrence status was marginally significant as well (*P* = 0.056, Table 2), which suggests that HPSE expression might be significantly correlated with recurrence if the sample size was big enough. No correlation was found between HPSE protein staining score and other clinical features by this analysis.

### The impact of HPSE mRNA and protein levels on recurrence and metastasis in HCC patients

The above analysis showed that there was a significant or marginally significant correlation between HPSE expression and recurrence or metastasis in chi-square test. We further explored whether HPSE expression (mRNA and protein levels) was a predictor for recurrence or metastasis in HCC patients using Kaplan-Meier curve, Log-rank test and Cox regression analysis. First, we conducted Kaplan-Meier curve analysis and Log-rank test, which showed that the group with lower mRNA level of HPSE had significantly higher cumulative metastasis rate and recurrence rate than the group with high expression level (Log-rank test, *P* = 0.033 and *P* = 0.023, respectively, Fig. 4A and 4B); Similarly, the group with lower protein level of HPSE had significantly higher cumulative recurrence rate than the group with high expression level (Log-rank test, *P* = 0.035, Fig. 4D). However, no difference was found in the cumulative metastasis rates between the groups with low or high level of HPSE protein (Log-rank test, *P* = 0.199, Fig. 4C), which is likely to be due to the relatively small number of metastasis (only 10 cases) and sample size (only 80 cases) analyzed by IHC.

Next, Cox regression analysis was conducted to find the independent predictors for recurrence and metastasis in HCC patients. The univariate Cox regression analysis on metastasis indicated that only HPSE mRNA level was significantly associated with metastasis (*P* = 0.045, Table S3), and that HPSE mRNA level, protein expression score and serum AFP concentration were significantly associated with recurrence (Table S4). With an Enter procedure, multivariate Cox regression analysis suggested that HPSE mRNA level and serum AFP were the independent predictors for recurrence in HCC (*P* = 0.031 and *P* = 0.046, respectively, Table 3).

Since the causes and mechanisms of early recurrence (within two years after hepatectomy) and late recurrence (in more than two years after hepatectomy) might be very different, we tested whether HPSE mRNA expression impacted the early or late recurrence in HCC patients. Kaplan-Meier plots and Log-rank test indicated that the patients with lower HPSE mRNA level had higher early recurrence rate (Log-rank test, *P* = 0.025, Fig. 5A), but no difference in the late recurrence rates between the groups with low or high level of HPSE mRNA (Fig. 5B). Both univariate and multivariate Cox regression models revealed that HPSE mRNA level and serum AFP were the significant predictors for early recurrence in HCC (*P* = 0.031 and *P* = 0.016, respectively in univariate analysis, Table S5; *P* = 0.020 and *P* = 0.012, respectively in multivariate analysis, Table 4;), and no predictor was identified for late recurrence in HCC (all *P*>0.05, Table S6).

### The impact of HPSE protein and mRNA levels on HCC patients' survival

To evaluate the importance of HPSE expression in patients' survival, we carried out the Kaplan-Meier analysis and Log-rank test on the gene's expression level. The median expression value of mRNA was used as the cut-off to separate patients into groups with high or low levels. Based on the protein expression score, patients also were divided into groups with high (score III or IV) or low level (score I or II). The analysis showed that the group with lower HPSE mRNA or protein level had significantly worse overall survival rate than did the group with high expression level (Log-rank test, *P* = 0.009, *P* = 0.024, for mRNA and protein, respectively; see Fig. 6A and 6B). This indicates that the reduced level of mRNA or protein of HPSE is strongly correlated with poor survival of HCC patients. We next performed univariate Cox regression analysis to find all of the factors with potential prognostic significance in HCC patients. The analysis demonstrated that mRNA level and protein staining score of HPSE were both significantly associated with overall survival rate (Table S7). The univariate analysis also indicated that clinical parameters including tumor size, tumor grade, number of tumor nodules and serum AFP were significantly associated with the overall survival rate (Table S7). Finally, multivariate Cox regression analysis was used to identify the independent prognostic predictors. With an Enter procedure, multivariate Cox regression analysis was implemented on all of the variables that showed prognostic significances in the univariate analysis. The result demonstrated that HPSE protein staining score, tumor size and nodule number were the independent predictors for overall survival in HCC (*P* = 0.041, *P* = 0.019, *P* = 0.002, respectively, Table 5).

### Stratified analysis of the role of HPSE mRNA and protein levels on survival in subgroups of BCLC stages

Survival analysis showed that HPSE mRNA and protein levels were associated with survival of HCC patients, but only protein expression was the independent predictor for HCC patients. To explore whether the HPSE mRNA or protein had different roles in the survivals of early stage and late stage of HCC, we performed stratified analysis for patients with BCLC stage 0-A and stage B–D (BCLC Staging system, 2010). Kaplan-Meier curve analysis and Log-rank test showed that in the subgroup of BCLC stage 0-A, patients with lower level of HPSE mRNA but not protein level had significantly worse overall survival rate than those with high expression level (Log-rank test, *P* = 0.008, *P* = 0.214, for mRNA and protein, respectively; see Fig. 7A and 7B); and in the subgroup of stage B–D, patients with lower level of HPSE protein but not mRNA level had significantly worse overall survival rate than those with high expression level (Log-rank test, *P* = 0.502, *P* = 0.004, for mRNA and protein, respectively; see Fig. 7C and 7D).

Cox regression analysis was next conducted to identify independent predictors for overall survival in the subgroup analysis. The univariate Cox regression analysis indicated that only HPSE mRNA level was significantly associated with overall survival in the subgroup of BCLC stage 0-A (*P* = 0.013, Table S8), and that HPSE protein expression score and tumor grade were significantly associated with overall survival in the subgroup of BCLC stage B–D (*P* = 0.011 and *P* = 0.022, respectively, Table S9). With an Enter procedure, multivariate Cox regression analysis suggested that only HPSE protein expression score was the independent predictor for overall survival in the subgroup of BCLC stage B–D (*P* = 0.020, Table 6).

## Discussion

As the mentioned above, we and others found a high frequency of LOH at D4S2964 in HCC. To identify the gene(s) involved, we performed another LOH analysis on 440 SNPs in 49 genes surrounding the D4S2964 locus in HCC by a custom SNP microarray and found that several genes had higher LOH rate [18]. In the present study, our result displayed a high frequency of LOH (average 31.6%) at 10 SNP loci located in the HPSE gene (Table 1). Further analyses showed that LOH at 3 SNPs in HPSE was correlated with serum AFP, HBV-DNA level and tumor stage. At the same time, we found HPSE DNA deletion in 38.74% HCC patients using real-time PCR. As expected, the reduced expression levels of HPSE mRNA and protein were observed in HCC tissues and associated with poor outcomes in HCC patients. Therefore, those results suggest that HPSE is affected by the LOH at D4S2964 and is a potential tumor suppressor gene.

The evidences obtained from this study are consistent with previous reports by other investigators. In 2002, Ikeguchi et al found that the mRNA level of HPSE detected in HCC was reduced when compared with corresponding non-tumor liver tissues [23]. These authors further reported that decreased HPSE gene expression was significantly associated with a poor disease-free survival of the HCC patients [24]. A significant positive correlation between HPSE expression level and apoptotic index of hepatocytes was also observed [24], which suggested that high level of HPSE expression might be associated with increased apoptosis of liver cells. In addition, they reported similar situations in esophageal squamous cell carcinoma [25]. These observations are in agreement with our present study, indicating that HPSE gene is a potential tumor suppressor gene in HCC.

On the other hand, several other studies showed the mRNA expression levels of HPSE gene were significantly higher in primary HCC tissues compared with the non-cancer tissues and/or normal controls, and the increased expression of HPSE mRNA was correlated with larger tumor size, poor tumor grade, portal vein invasion, tumor microvessel density, and post-operative metastasis in HCC [26], [27]. These reports are inconsistent with the results from Ikeguchi and the present study. However, the patient number in most of these studies was significantly less (fewer than 56 cases in contrast to 112 cases in our study). Therefore, our result is deemed to be more reliable.

With respect to the conflicting report on HPSE in HCC, Dong and Wu had discussed possible reasons for the contradictory results obtained from different HCC studies [12]. They speculated that the inconsistent results from different reports on HPSE in HCC might be resulted from the different protein subcellular locations (nucleus, cytoplasm and cell surface), expression levels (low, moderate and high) and activities (enzymatic and non-enzymatic activities) of HPSE because it could have different effects in different sites, expression levels and activities [12]. It should be mentioned here that HPSE protein was located in both the cytoplasm and nucleus of liver cancer cell in the present study, and the protein in the nucleus was reported to be favorable to the outcome of cancer [9], [11]. We also noticed that 12.6% of patients had HPSE DNA copy number gain (>2.24), which suggested that HPSE gene not only was deleted, but also occasionally amplified. However, the clinical significance of the small number of DNA copy number gain was not clear in this study. In addition, HPSE transcript has different splice variants, which possessed different functions from the wild one [28], [29]. For example, HPSE splice variant 6 from *Spalax* showed inhibition of HS degradation, suppression of tumor growth and no enzymic activity, while splice variant 7 could enhanced tumor growth but had no enzymatic activity [28]. Conceivably, one of the reasons causing the conflicting results might be that different splice variants of HPES were detected in different reports. These factors may explain the contrary reports on HPSE gene in HCC and other cancers.

For more than twenty years, HPSE was thought to function as an oncogene and regarded as a therapeutic target [30]. Consequently, many HPSE inhibitors including chemically modified natural products, small molecule inhibitors, sugar and Neutralizing antibodies, have been developed with some tested in clinical trials [30], [31]. However, none of the HPSE inhibitors has showed favorable clinical outcome. For example, PI-88, a sugar inhibitor for HPSE, had been tested in phase I/II clinical trials for melanoma, prostate cancer and HCC [32], [33], [34], but no significant improvement for patients had been observed. In fact, HCC patients with higher PI-88 dose (250 mg/day) even had worse outcome in the phase II clinical trial [32].

The poor therapeutic effects with HPSE inhibitors beg for a fresh thinking on the role of HPSE in tumor progression. Many studies pointed to the fact that HPSE is not simply an oncogene because of its complex biological functions. More importantly, Zetser et al observed that high HPSE expression level could inhibit tumor growth [35]. Nobuhisa et al proposed that translocating HPSE protein into cell nucleus could be as a new strategy for anti-cancer therapy, which was based on their finding that HPSE protein localized in the nucleus of HL-60 cell nucleus caused cell differentiation [36]. The evidences from these studies and our present study support our hypothesis that HPES can serve as a tumor suppressor gene. Accordingly, it is prudent that caution be taken for HSPE inhibitors as anticancer therapeutics until more clear evidence is available regarding HPSE's biological functions and clinical significances.

## Supporting Information

Table S1 The primer sequences for quantitative RT-PCR assay.
(DOC)

Table S2 LOH frequencies and mRNA expressions of 11 genes and their relationships with prognosis.
(DOC)

Table S3 Univariate Cox regression analysis of variables affecting metastasis.
(DOC)

Table S4 Univariate Cox regression analysis of impacts of variables affecting recurrence.
(DOC)

Table S5 Univariate Cox regression analysis of variables affecting early recurrence.
(DOC)

Table S6 Univariate Cox regression analysis of variables affecting late recurrence.
(DOC)

Table S7 Univariate Cox regression analysis of variables affecting overall survival.
(DOC)

Table S8 Univariate Cox regression analysis of variables affecting overall survival in the subgroup of BCLC stage 0, A.
(DOC)

Table S9 Univariate Cox regression analysis of variables affecting overall survival in the subgroup of BCLC stage B, C, D.
(DOC)
