# Supplementary material for: Allele Loss and Down-Regulation of Heparanase Gene Are Associated with the Progression and Poor Prognosis of Hepatocellular Carcinoma
Source: PLoS One. 2012 Aug 31;7(8):e44061. doi: 10.1371/journal.pone.0044061 (PMC3432106; doi:10.1371/journal.pone.0044061)
Supplement: Table S1 — The primer sequences for quantitative RT-PCR assay. (DOC) [file pone.0044061.s001.doc]

| **Table S1. The primer sequences for quantitative RT-PCR assay** | | |
| --- | --- | --- |
| Gene | Forward primer | Reverse primer |
| PPEF2 | GGAACAACTGAGTCGCGAGAAC | CCAGTGAGATGAACCCTGAATGAT |
| PRDM8 | GGTACGGGAAAGAACTGACTGA | CGCAGATGCGCCACATAG |
| SDAD1 | AAGTTGGAAAAGGCAATGAAAGTG | TCCGCAAAATCTTGGGGATC |
| CXCL9 | CCTTAAACAATTTGCCCCAAGC | GGCTGACCTGTTTCTCCCACTT |
| CCDC158 | TGTGCCATAGAAGCAACAACTCG | TGTGAATGTGAAGCAACCAGAGG |
| PRKG2 | TTCTGGGGTTGACCAAATGATG | GCCTGCAAAGCCTCCGAAT |
| HPSE | CTGATGTTGGTCAGCCTCGAAG | TGGTAGCAGTCCGTCCATTCAA |
| AGPAT9 | ACGCTGGTTCTCGGCTTCAT | TCGACTCCTTTGGGGTTCCTT |
| HELQ | AAAGGTCTTGAAGGACTTGTGC | GAGTTGGCTAAACTGCCTGAAG |
| NUP54 | CTCAGTTCAAGGGCCGACT | AATGCTGCTTGATTTCTCGTAA |
| CCNG2 | TGAGGCTACCCCGGAGAATG | AAGAGCCAAGAACCTGTCCAAAAT |
| GAPDH | CTCCTCCTGTTCGACAGTCAGC | CCCAATACGACCAAATCCGTT |
