# Supplementary material for: Allele Loss and Down-Regulation of Heparanase Gene Are Associated with the Progression and Poor Prognosis of Hepatocellular Carcinoma
Source: PLoS One. 2012 Aug 31;7(8):e44061. doi: 10.1371/journal.pone.0044061 (PMC3432106; doi:10.1371/journal.pone.0044061)
Supplement: Table S2 — LOH frequencies and mRNA expressions of 11 genes and their relationships with prognosis. (DOC) [file pone.0044061.s002.doc]

| **Table S2. LOH frequency and mRNA expression of 11 genes and their relationships with Prognosis** | | | | | | |
| --- | --- | --- | --- | --- | --- | --- |
| No: | Gene | **Gene LOH** | **mRNA level in 50 cases** | | **mRNA level in 112 cases** | |
|  |  | % (LOH/inf)^a^ | Under-expressed in tumor tissues, % (n) | Relationship with poor survival (*P* value)^b^ | Under-expressed in tumor tissues, % (n) | Relationship with poor survival (*P* value)^b^ |
| 1 | PPEF2 | 64.3% (9/14) | 82.0% (41) | 0.078 | 82.1% (92) | 0.650 |
| 2 | PRDM8 | 51.8% (43/83) | 80.0% (40) | 0.540 | - - | - - |
| 3 | SDAD1 | 41.5% (39/94) | 80.0% (40) | 0.145 | - - | - - |
| 4 | CXCL9 | 52.9% (45/85) | 76.0% (38) | 0.907 | - - | - - |
| 5 | CCDC158 | 45.5% (25/55) | 80.0% (40) | 0.045 | 80.4% (90) | 0.014 |
| 6 | CCNG2 | 27.0% (17/63) | 60.0% (30) | 0.023 | 56.3% (63) | 0.016 |
| 7 | PRKG2 | 40.4% (38/94) | 80.0% (40) | 0.157 | - - | - - |
| 8 | HPSE | 48.2% (41/85) | 80.0% (40) | 0.019 | 71.4% (80) | 0.009 |
| 9 | AGPAT9 | 60.4% (58/96) | 90.0% (45) | 0.008 | 84.8% (95) | 0.020 |
| 10 | HELQ | 50.6% (41/81) | 90.0% (45) | 0.446 | - - | - - |
| 11 | NUP54 | 51.1% (47/92) | 76.0% (38) | 0.514 | - - | - - |
| a: LOH, Case number with gene LOH; inf, informative case number.  b: Survival function was performed with Kaplan-Meier curve and log-rank test. | | | | | | |
