# Supplementary material for: Allele Loss and Down-Regulation of Heparanase Gene Are Associated with the Progression and Poor Prognosis of Hepatocellular Carcinoma
Source: PLoS One. 2012 Aug 31;7(8):e44061. doi: 10.1371/journal.pone.0044061 (PMC3432106; doi:10.1371/journal.pone.0044061)
Supplement: Table S3 — Univariate Cox regression analysis of variables affecting metastasis. (DOC) [file pone.0044061.s003.doc]

| **Table S3.** **Univariate Cox regression analysis of variables affecting metastasis** | | | |
| --- | --- | --- | --- |
| Parameter | Hazard ratio | Confidence interval (95%) | *P* value |
| HPSE mRNA level | 3.290 | 1.029 - 10.521 | 0.045 |
| HPSE protein score | 2.658 | 0.563 - 12.546 | 0.217 |
| Sex | 1.244 | 0.278 - 5.562 | 0.775 |
| Tumor grade | 2.171 | 0.798 - 5.908 | 0.129 |
| Serum HBsAg | 2.367 | 0.309 - 18.101 | 0.407 |
| Serum AFP | 3.153 | 0.705 - 14.102 | 0.133 |
| Tumor size | 5.225 | 0.682 - 40.039 | 0.112 |
| No. of nodules | 0.340 | 0.044 - 2.621 | 0.300 |
| Cirrhosis | 1.737 | 0.227 - 13.288 | 0.595 |
