# Supplementary material for: Allele Loss and Down-Regulation of Heparanase Gene Are Associated with the Progression and Poor Prognosis of Hepatocellular Carcinoma
Source: PLoS One. 2012 Aug 31;7(8):e44061. doi: 10.1371/journal.pone.0044061 (PMC3432106; doi:10.1371/journal.pone.0044061)
Supplement: Table S4 — Univariate Cox regression analysis of impacts of variables affecting recurrence. (DOC) [file pone.0044061.s004.doc]

| **Table S4.** **Univariate Cox regression analysis of impacts of variables affecting recurrence** | | | |
| --- | --- | --- | --- |
| Parameter | Hazard ratio | Confidence interval (95%) | *P* value |
| HPSE mRNA level | 2.122 | 1.088 - 4.138 | 0.027 |
| HPSE protein score | 2.528 | 1.027 - 6.222 | 0.043 |
| Sex | 1.142 | 0.444 - 2.936 | 0.783 |
| Tumor grade | 1.575 | 0.864 - 2.871 | 0.138 |
| Serum HBsAg | 2.041 | 0.626 - 6.659 | 0.237 |
| Serum AFP | 2.772 | 1.156 - 6.651 | 0.022 |
| Tumor size | 1.865 | 0.775 - 4.491 | 0.164 |
| No. of nodules | 1.635 | 0.783 - 3.411 | 0.190 |
| Cirrhosis | 4.987 | 0.681 - 36.516 | 0.114 |
