# Supplementary material for: Allele Loss and Down-Regulation of Heparanase Gene Are Associated with the Progression and Poor Prognosis of Hepatocellular Carcinoma
Source: PLoS One. 2012 Aug 31;7(8):e44061. doi: 10.1371/journal.pone.0044061 (PMC3432106; doi:10.1371/journal.pone.0044061)
Supplement: Table S5 — Univariate Cox regression analysis of variables affecting early recurrence. (DOC) [file pone.0044061.s005.doc]

| **Table S5.** **Univariate Cox regression analysis of variables affecting early recurrence** | | | |
| --- | --- | --- | --- |
| Parameter | Hazard ratio | Confidence interval (95%) | *P* value |
| HPSE mRNA level | 2.257 | 1.079 - 4.720 | 0.031 |
| HPSE protein score | 1.877 | 0.744 - 4.734 | 0.182 |
| Sex | 1.211 | 0.423 - 3.465 | 0.721 |
| Tumor grade | 1.602 | 0.838 - 3.064 | 0.154 |
| Serum HBsAg | 1.599 | 0.486 - 5.261 | 0.440 |
| Serum AFP | 3.617 | 1.265 - 10.344 | 0.016 |
| Tumor size | 1.736 | 0.665 - 4.533 | 0.260 |
| No. of nodules | 1.919 | 0.898 - 4.102 | 0.092 |
| Cirrhosis | 24.983 | 0.211 - 2.959×10^3^ | 0.186 |
