# Supplementary material for: Allele Loss and Down-Regulation of Heparanase Gene Are Associated with the Progression and Poor Prognosis of Hepatocellular Carcinoma
Source: PLoS One. 2012 Aug 31;7(8):e44061. doi: 10.1371/journal.pone.0044061 (PMC3432106; doi:10.1371/journal.pone.0044061)
Supplement: Table S6 — Univariate Cox regression analysis of variables affecting late recurrence. (DOC) [file pone.0044061.s006.doc]

| **Table S6.** **Univariate Cox regression analysis of variables affecting late recurrence** | | | |
| --- | --- | --- | --- |
| Parameter | Hazard ratio | Confidence interval (95%) | *P* value |
| HPSE mRNA level | 1.569 | 0.310 - 7.938 | 0.586 |
| HPSE protein score | 55.319 | 0.039 - 7.772×10^4^ | 0.278 |
| Sex | 0.866 | 0.100 - 7.491 | 0.896 |
| Tumor grade | 1.425 | 0.292 - 6.968 | 0.662 |
| Serum HBsAg | 28.430 | 0.004 - 2.272×10^5^ | 0.465 |
| Serum AFP | 1.079 | 0.197 - 5.917 | 0.930 |
| Tumor size | 2.567 | 0.298 - 22.140 | 0.391 |
| No. of nodules | 0.039 | 0.000 - 2.526×10^3^ | 0.567 |
| Cirrhosis | 0.701 | 0.078 - 6.319 | 0.752 |
