# Supplementary material for: Allele Loss and Down-Regulation of Heparanase Gene Are Associated with the Progression and Poor Prognosis of Hepatocellular Carcinoma
Source: PLoS One. 2012 Aug 31;7(8):e44061. doi: 10.1371/journal.pone.0044061 (PMC3432106; doi:10.1371/journal.pone.0044061)
Supplement: Table S7 — Univariate Cox regression analysis of variables affecting overall survival. (DOC) [file pone.0044061.s007.doc]

| **Table S7.** **Univariate Cox regression analysis of variables affecting overall survival** | | | |
| --- | --- | --- | --- |
| Parameter | Hazard ratio | Confidence interval (95%) | *P* value |
| HPSE mRNA level | 2.368 | 1.209 - 4.637 | 0.012 |
| HPSE protein score | 2.674 | 1.096 - 6.527 | 0.031 |
| Sex | 1.327 | 0.518 - 3.398 | 0.556 |
| Tumor grade | 1.419 | 1.005 - 2.003 | 0.046 |
| Serum HBsAg | 0.902 | 0.377 - 2.158 | 0.817 |
| Serum AFP | 3.319 | 1.295 - 8.504 | 0.012 |
| Tumor size | 4.266 | 1.310 - 13.888 | 0.016 |
| No. of nodules | 3.143 | 1.627 - 6.073 | 0.001 |
| Cirrhosis | 0.937 | 0.329 - 2.673 | 0.903 |
