# Supplementary material for: Allele Loss and Down-Regulation of Heparanase Gene Are Associated with the Progression and Poor Prognosis of Hepatocellular Carcinoma
Source: PLoS One. 2012 Aug 31;7(8):e44061. doi: 10.1371/journal.pone.0044061 (PMC3432106; doi:10.1371/journal.pone.0044061)
Supplement: Table S8 — Univariate Cox regression analysis of variables affecting overall survival in the subgroup of BCLC stage 0, A. (DOC) [file pone.0044061.s008.doc]

| **Table S8．Univariate Cox regression analysis of variables affecting overall survival** | | | |
| --- | --- | --- | --- |
| **in the subgroup of BCLC stage 0,A** | | | |
| Parameter | Hazard ratio | Confidence interval (95%) | *P* value |
| HPSE mRNA level | 3.641 | 1.309 - 10.128 | 0.013 |
| HPSE protein score | 2.195 | 0.612 - 7.871 | 0.228 |
| Sex | 1.503 | 0.347 - 6.506 | 0.586 |
| Tumor grade | 1.368 | 0.606 - 3.090 | 0.450 |
| Serum HBsAg | 0.960 | 0.280 - 3.296 | 0.948 |
| Serum AFP | 2.967 | 0.864 - 10.187 | 0.084 |
| Tumor size | 6.861 | 0.915 - 51.477 | 0.061 |
| No. of nodules | 0.043 | 0.000 - 102.648 | 0.428 |
| Cirrhosis | 1.534 | 0.202 - 11.620 | 0.679 |
