# Supplementary material for: Allele Loss and Down-Regulation of Heparanase Gene Are Associated with the Progression and Poor Prognosis of Hepatocellular Carcinoma
Source: PLoS One. 2012 Aug 31;7(8):e44061. doi: 10.1371/journal.pone.0044061 (PMC3432106; doi:10.1371/journal.pone.0044061)
Supplement: Table S9 — Univariate Cox regression analysis of variables affecting overall survival in the subgroup of BCLC stage B, C, D. (DOC) [file pone.0044061.s009.doc]

| **Table S9．Univariate Cox regression analysis of variables affecting overall survival in the subgroup of BCLC stage B,C,D** | | | |
| --- | --- | --- | --- |
| Parameter | Hazard ratio | Confidence interval (95%) | *P* value |
| HPSE mRNA level | 1.365 | 0.543 - 3.429 | 0.508 |
| HPSE protein score | 7.275 | 1.569 - 33.741 | 0.011 |
| Sex | 1.716 | 0.497 - 5.929 | 0.393 |
| Tumor grade | 2.795 | 1.161 - 6.729 | 0.022 |
| Serum HBsAg | 0.650 | 0.186 - 2.271 | 0.500 |
| Serum AFP | 2.468 | 0.569 - 10.713 | 0.228 |
| Tumor size | 2.646 | 0.610 - 11.476 | 0.194 |
| No. of nodules | 1.821 | 0.512 - 6.478 | 0.355 |
| Cirrhosis | 1.428 | 0.406 - 5.026 | 0.579 |
